# Long-term tumour control in sacral chordoma following high-dose palliative image-guided intensity-modulated radiotherapy (IG-IMRT)

**DOI:** 10.1259/bjrcr.20160145

**Published:** 2017-04-12

**Authors:** Amy Jackson, Sarah Scott, Marina Romanchikova, David J Noble, Neil G Burnet

**Affiliations:** ^1^Oncology Centre, Addenbrooke's Hospital, Cambridge University Hospitals NHS Foundation Trust, Cambridge, UK; ^2^Department of Medical Physics and Clinical Engineering, Addenbrooke’s Hospital, Cambridge, UK; ^3^Department of Oncology, University of Cambridge, Cambridge Biomedical Campus, Addenbrooke's Hospital, Cambridge, UK

## Abstract

Chordomas can present a challenge to the radiation oncologist and surgeon owing to the proximity to neurological structures. We describe a case of long-term tumour control in a chordoma of the lumbar spine following high-dose palliative radiotherapy. Image-guided intensity-modulated radiotherapy with photons provided a good solution to deliver 65 Gy to the tumour in a technically challenging case, and local control has been sustained over a period of years.

## Background

Chordomas are rare malignant tumours thought to arise from the embryonic notochord. Management is complex because of their anatomical location and relative radioresistance. About 30–40 cases of chordoma present in the UK each year. This rarity adds to the challenge of developing effective management strategies. They occur most commonly in the skull base (35%) and sacrum (50%), but tumours can arise along the whole length of the spine.^1^ Radiotherapy treatment of chordoma is complicated by the proximity of dose-limiting tissues and the metal implants often required for reconstruction following surgical resection.^2^

The typical growth rate of chordoma is slow. The risk of metastasis may be as high as 30%,^2^ with additional risk of seeding along surgical tracks. It can be difficult to distinguish between chordoma and chondrosarcoma with imaging. Histologically, classical chordomas have characteristic physaliphorous (“bubble bearing”) cells, which are the result of prominent intracellular vacuoles.^2^ Chordomas contain extracellular mucin, giving high *T*_2_ signal on MRI that can be used to assess tumour extent.

While there is good evidence that chordoma has a dose response to radiation treatment,^3^ the TD_50_ (the dose required to achieve local control in 50% of tumours) is around 65 Gy, regardless of the modality. This is relatively high and radiotherapy dose delivery is difficult to achieve without exceeding conventional normal tissue constraints. The most successful series have used proton beam therapy (PBT) or carbon ion therapy.^2^ High-dose X-ray treatments have also been reported as useful.^4^

With charged particle therapy as the preferred choice for curative treatment, the multidisciplinary management of recurrent chordomas remains challenging. We report the case of a patient treated with image-guided intensity-modulated radiotherapy (IG-IMRT) using X-rays for a large locally recurrent lumbar chordoma, with metastases along the surgical track, who has durable tumour control at 6 years.

## Clinical presentation and initial treatment

**Figure 1.**
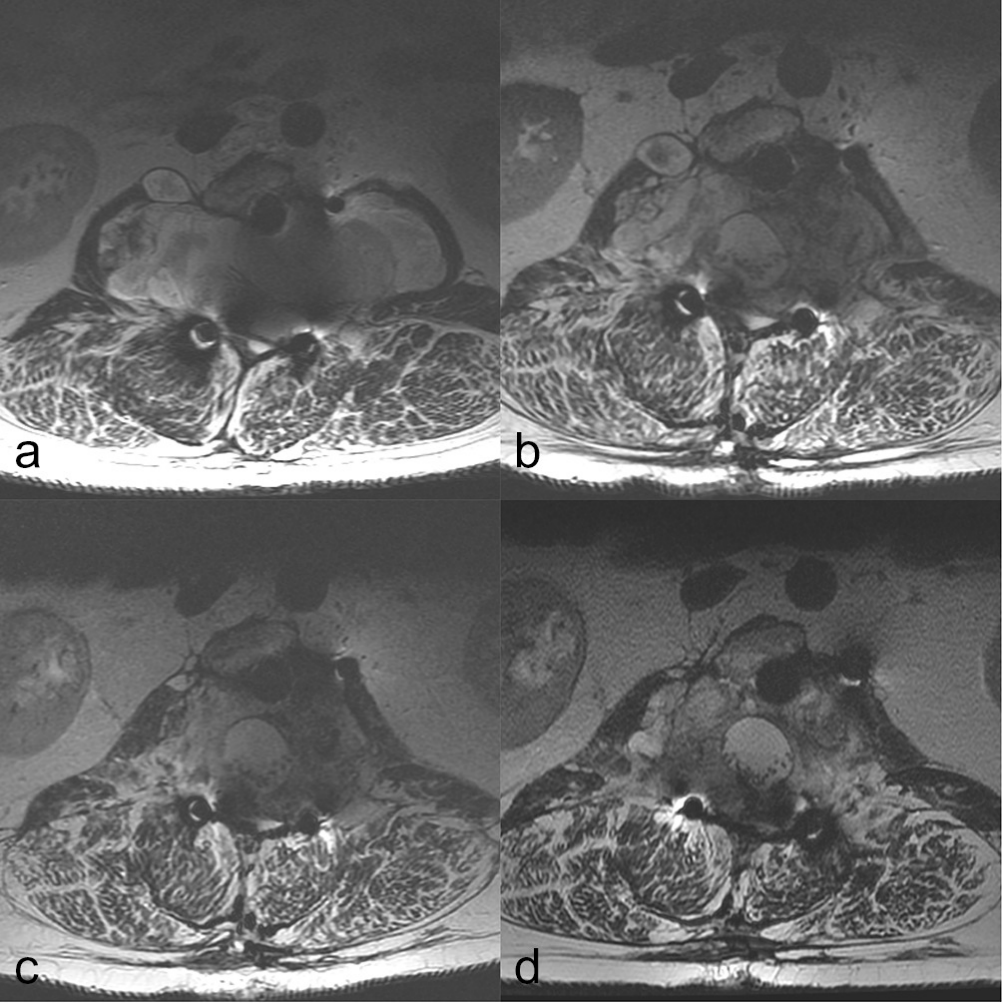
*T*_2_ weighted MRI scans at the level of L2. Note the artefact caused by the metal reconstruction, which consists of a central vertebral cage and posterior rods. (a) Pre-radiotherapy (in 2010). A large mass is seen centred on the spine and involving the psoas muscles bilaterally. Tumour involves the body of the vertebra. A separate nodule is seen on the right psoas anteriorly. The whole spinal canal is filled with tumour and the nerve roots cannot be resolved separately. (b) Year 1 post-radiotherapy, (c) Year 3 post- radiotherapy, (d) Year 5 post-radiotherapy. Substantial reduction in the size of the mass, and the lumbar nerve roots can now be seen.

A 50-year-old male was referred in 2010 for consideration of palliative radiotherapy for uncontrolled chordoma recurrence. Symptoms had started 7 years earlier in 2003, with unilateral leg pain. Subsequent investigation demonstrated a 10 × 4 cm mass at L2, considered likely to be a chordoma. A two-stage surgical resection with internal metal fixation was performed in 2005. Post-operative histology confirmed chordoma, and complete resection with adequate margins was achieved. The disease was controlled for 3 years, but in 2008 pain returned, with paraesthesia in the upper leg and some alteration in bladder sensation. Repeat imaging at this point confirmed local recurrence with deposits of disease along the previous surgical track. A debulking procedure was performed but complete resection was not possible. Post-operative MRI following this showed reduction in the left side of the chordoma mass. However, there was bulky disease on the right side adjacent to L2, with superior and inferior satellite nodules. These tracked down the previous surgical approaches into the psoas muscle (Figure 1a). The patient was referred for consideration of PBT. This was not recommended since the disease was considered incurable, and because of concerns about outcomes of PBT in the presence of metal spinal reconstruction.^5^ The tumour increased slowly in size over the next 2 years and further surgery was not possible. Tumour around the conus medullaris and lumbosacral nerve roots suggested that neurological damage was imminent.

In 2010 the patient was referred for consideration of IG-IMRT using TomoTherapy. Symptoms were limited, with mild alteration in bladder sensation, and no reserve of bladder control. Well-healed surgical scars were visible, and movement in the low back was limited. The objective of the treatment was palliative, specifically aiming to delay neurological damage including loss of sphincter control.

## Radiotherapy treatment

Radiotherapy was delivered using the TomoTherapy HiArt system. The 6 megaelectron voltage (MeV) treatment beam is delivered through full 360 degrees, approximating to 51 different beam directions.^6^ Helical dose delivery permits treatment fields up to 160 cm in length, although only 35 cm was used in this case. This system can achieve high degrees of modulation, ideal for treating complex shaped targets adjacent to critical organs.^7^

Metalwork artefact obscured the planning kilovoltage CT so a dedicated megavoltage CT (MVCT) scan was performed and co-registered with the kilovoltage CT to aid target volume delineation. MV X-ray tissue interactions are dominated by Compton scattering, which means this artefact does not affect CT image quality and improves image guidance. The main tumour, satellite nodules and organs at risk (OARs) were outlined on the planning CT scan, aided by the diagnostic MRI and MVCT scans. There was extensive tumour recurrence around the lumbar region with encased lumbar nerve roots. Areas between the satellite nodules were deemed at high risk of microscopic spread, and so were included in the clinical target volume. A 3 mm margin was added to form the planning target volume (PTV). The main objective was to deliver 65 Gy or more to the primary PTV in 39 daily fractions. It was hoped that a 70 Gy boost could be delivered to the primary gross tumour volume (GTV), with 67–68 Gy to the satellite nodule GTVs. This was not possible because of dose limitations from the surrounding normal tissue structures.

The planning dose constraints to the OARs, based on the QUANTEC guidance, are outlined in Table 1.^8^ Constraining spinal cord dose is critical to prevent long-term injury. The contoured spinal cord was extended by 2 mm axially and 0 mm longitudinally to form a planning organ at risk volume (PRV). As the spinal cord is a serial organ, a maximum dose of 58.6 Gy in 39 fractions to the spinal cord PRV was used as a constraint. Assuming an alpha:beta ratio of 2, this is biologically equivalent to 54 Gy in 30 fractions.

**Table 1.** Planning dose objectives and constraints for target and organs at risk

Region of interest	Objective	Absolute dose constraint
Primary GTV	≥ 70 Gy	
Primary PTV—cord PRV	≥ 65 Gy	
Secondary nodule GTVs	≥ 67 Gy	
Spinal cord PRV		58.6 Gy
Sacral nerve roots		70 Gy
Kidney V_24 Gy_	< 30%	
Kidney mean dose	< 18 Gy	20 Gy
Liver V_24 Gy_	< 5%	
Liver V_30 Gy_		10%
Small bowel V_50 Gy_		10%
Large bowel V_50 Gy_	0%	15%
Stomach V_30 Gy_		20%
Ureters		65 Gy

GTV, gross tumour volume; PTV, planning target volume; PRV, planning organ at risk volume; V_x Gy_, Volume of organ receiving x Gy.

Dose constraints to the spinal cord and also the lumbar nerve roots were met. In contrast, the kidneys are parallel organs; thus mean dose and volume receiving more than 24 Gy (V_24Gy_) are more predictive of long-term toxicity. In this case the V_24Gy_ objective was met, but the mean kidney dose was higher than hoped at 19 Gy. The liver V_24Gy_ was not met owing to the proximity of the primary tumour to the liver.

**Figure 2.**
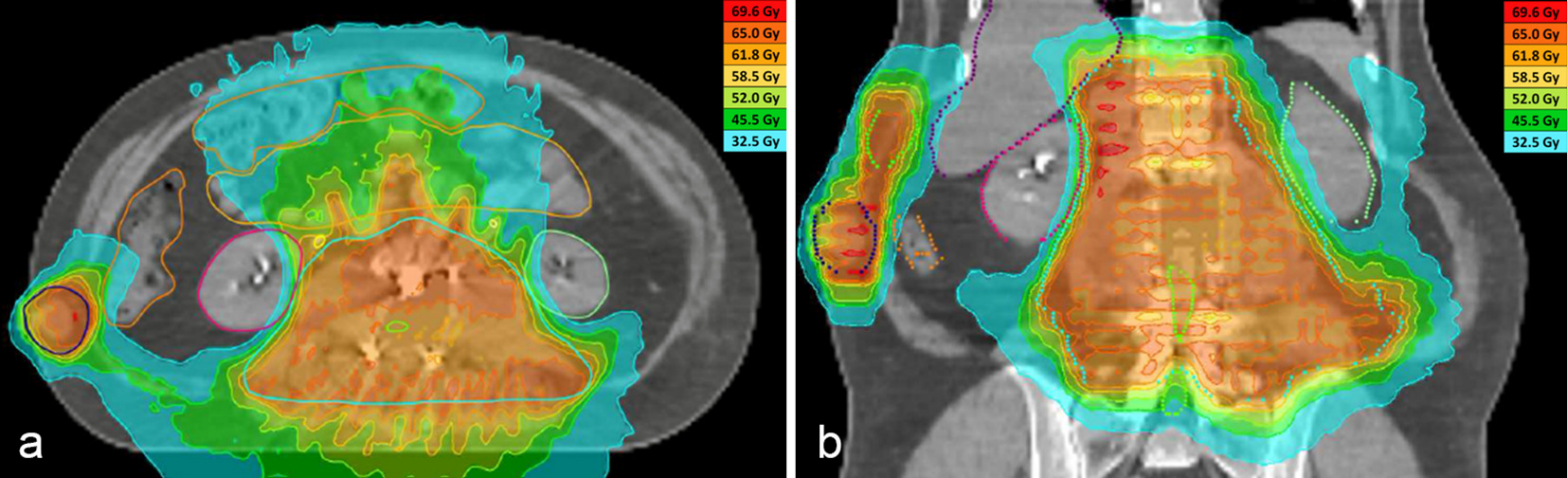
A dose distribution of plan in axial (a) and coronal (b) planes. The main planning target volume (PTV) (pale blue) and PTVs for two tumour nodules (dark blue & green) are shown. Within the main PTV, three components of the gross tumour volume are seen (2 blue/purple in a, and pink in b). Contours of both kidneys, liver, large bowel, small bowel, ureters (yellow), and planning organ at risk volumes for both ureters are shown. In (b), the approximate position of the nerve roots below the conus medularis has been contoured in green within the PTV. Lower dose isodoses are turned off for clarity.

Figure 2 illustrates the dose distribution. Areas of the PTV that received only 90% of the prescribed dose are seen, although the median dose actually delivered to the GTV was 65 Gy. Dose coverage was also assessed using the generalized equivalent uniform dose (gEUD) approach.^9^ EUD was calculated for the target volumes, including for PTV minus cord PRV, which received 59.4 Gy (Table 2). This was considered acceptable in the context of a palliative treatment designed to prevent neurological deterioration.

**Table 2.** Equivalent uniform doses to the target structures

Volume	EUD (Gy)
GTV main	65.4
GTV nodule 1	64.4
GTV nodule 2	63.7
CTV main	63.7
CTV nodule 1	63.8
CTV nodule 2	62.3
PTV main–PRV cord	59.4
PTV nodule R1	62.7
PTV nodule R2	59.6

GTV, gross tumour volume; CTV, clinical target volume; PTV, planning target volume; PRV, planning organ at risk volume.

## Treatment plan delivery

A field width of 0.5 cm, with a pitch of 0.287, was used. The treatment field length was 31 cm, and the beam-on time 365.6 s. Daily image guidance MVCT was performed, with positional correction using a zero action level. Details of the daily positional corrections, which are normally ‘hidden’ in the proprietary archive, were extracted using our bespoke software and are shown in Table 3.

**Table 3.** Daily positional corrections in each direction, with the 3-dimensional shift calculated for each day

	Mean	Standard deviation	Range
Lateral (mm)	2.73	4.42	[–6.10; 10.60]
Longitudinal (mm)	3.95	3.38	[–9.50; 9.80]
Anterior-posterior (mm)	3.30	2.12	[–1.40; 7.40]
3-dimensional (mm)	7.89	2.67	[3.41; 14.86]
Rotation (degrees)	–0.12	0.53	[–1.7; 1.0]

## Outcome and follow-up

Six years after completion of radiotherapy, the patient was stable with no progressive neurological symptoms or features of metastatic disease. Pre-treatment disease-related symptoms including back pain and leg swelling had slightly improved, and there was no long-term toxicity from radiotherapy. Follow up-MRI (Figure 1b–d) showed tumour regression, and an un-irradiated satellite lesion was stable, raising the intriguing possibility of an abscopal effect.

## Discussion

This case illustrates that high-dose palliative radiotherapy with photons may provide long-term tumour control in locally advanced chordomas. A previous case series of chordomas and chondrosarcomas from our institution treated with X-rays, including some patients with metal reconstructions, has shown 5-year local control of 83% for patients receiving at least 65 Gy in 39 fractions.^4^ A PTV of less than 90 cm^3^ and a GTV less than 30 cm^3^ were predictive for local control. Dose homogeneity using EUD has also been shown to predict good local control.^10^ A value of –15 was used for the “a” exponent in the calculation of EUD [Prof A. Niermerko, personal communication]. This has been calculated for the GTV, CTV and PTV for our patient (Table 2). The EUD for the PTV minus PRV was below 65 Gy, even though the median GTV dose achieved was 65 Gy. This reflects the higher contribution of low-dose values, for example, close to OARs, to the EUD when an “a”-value of –15 is used, and may represent a prognostic assessment of the target dose.^10^

Using TomoTherapy, the onboard MVCT scan enables daily image guidance and correction for positional errors,^7^ with reduction in PTV margins. Without this, substantial positional variation would have been seen, and normal tissue doses would have been higher. The use of accurate IG-IMRT may have contributed to the long-term tumour control for our case. Charged particle therapy with protons would have been challenging in this case because of the extensive metalwork and its use is not standard practice. In some centres, a significant correlation between local control and the use of protons in the presence of metalwork is not seen.^11^ Outcomes using PBT may be worse in the presence of extensive metal reconstruction.^5^

This case is noteworthy for two reasons. The tumour recurrence was extensive and the volume of the main PTV was more than 3 l (3347 cm^3^). Secondly, as the tumour was close to the spinal cord and lumbosacral nerve roots, there were areas of low dose within the PTV. In fact, the EUD to the PTV–PRV cord was only 59.4 Gy. These two observations would have predicted a poor outcome for the patient, but instead the tumour regressed considerably. The case illustrates how IG-IMRT with photons can be used to deliver long-term tumour control for recurrent chordoma, even in a palliative setting, and should not be ignored as a possible treatment approach.

## Acknowledgments

We are grateful to the patient for his sustained encouragement for us to write this report and for providing his written consent. We are also grateful to Prof RE Taylor and Dr DWO Tilsley for referring the patient and for help in his ongoing care. We are grateful to Jane Sales & Mala Jayasundera for help in preparing the manuscript.

## Learning points

1. X-ray radiotherapy can be used to control recurrent chordomas, provided that high dose is delivered using image-guided IMRT.
2. Photons can be used effectively when charged particle therapy with protons is contraindicated or not possible.
3. MVCT scans can assist target volume delineation in the presence of metal reconstruction.
4. Treatment of bulk disease in this case appears to have arrested growth at both primary site and in an untreated tumour nodule.

## Consent

Written informed consent for publication was obtained from the patient.
